# Supplementary material for: Genotypes and phenotypes of G6PD deficiency among Indonesian females across diagnostic thresholds of G6PD activity guiding safe primaquine therapy of latent malaria
Source: PLoS Negl Trop Dis. 2021 Jul 16;15(7):e0009610. doi: 10.1371/journal.pntd.0009610 (PMC8318249; doi:10.1371/journal.pntd.0009610)
Supplement: S1 Table — (DOCX) [file pntd.0009610.s001.docx]

**S1 Table. Performance of CSG G6PD Test against reference test Pointe Scientific quantitative assay**

|  | **10% cut-off** | **30% cut-off** | **70% cut-off** | **80% cut-off** |
| --- | --- | --- | --- | --- |
| Trinity cutoff value (U/g Hb) | 1.104 | 3.313 | 7.729 | 8.833 |
| Number of samples with G6PD levels less than cut-off | 8 | 31 | 231 | 375 |
| Sensitivity percentage (95% CI) | 87.5 (47.4-99.7) | 80.7 (62.5-92.6) | 25.1 (19.7-31.2) | 17.2 (13.5-21.4) |
| Specificity percentage (95% CI) | 94.5 (93.4-95.5) | 95.4 (94.3-96.3) | 96.8 (95.8-97.6) | 96.9 (95.9-97.7) |
| PPV percentage (95% CI) | 35.8 (28.8-43.5) | 38.0 (31.9-44.4) | 21.4 (16.2-27.7) | 16.1 (11.9-21.5) |
| NPV percentage (95% CI) | 99.5 (97.2-99.93) | 99.3 (98.6-99.7) | 97.4 (97.2-97.5) | 97.1 (96.9-97.2) |
