# Supplementary figures and images for: Genotypes and phenotypes of G6PD deficiency among Indonesian females across diagnostic thresholds of G6PD activity guiding safe primaquine therapy of latent malaria
Source: PLoS Negl Trop Dis. 2021 Jul 16;15(7):e0009610. doi: 10.1371/journal.pntd.0009610 (PMC8318249; doi:10.1371/journal.pntd.0009610)

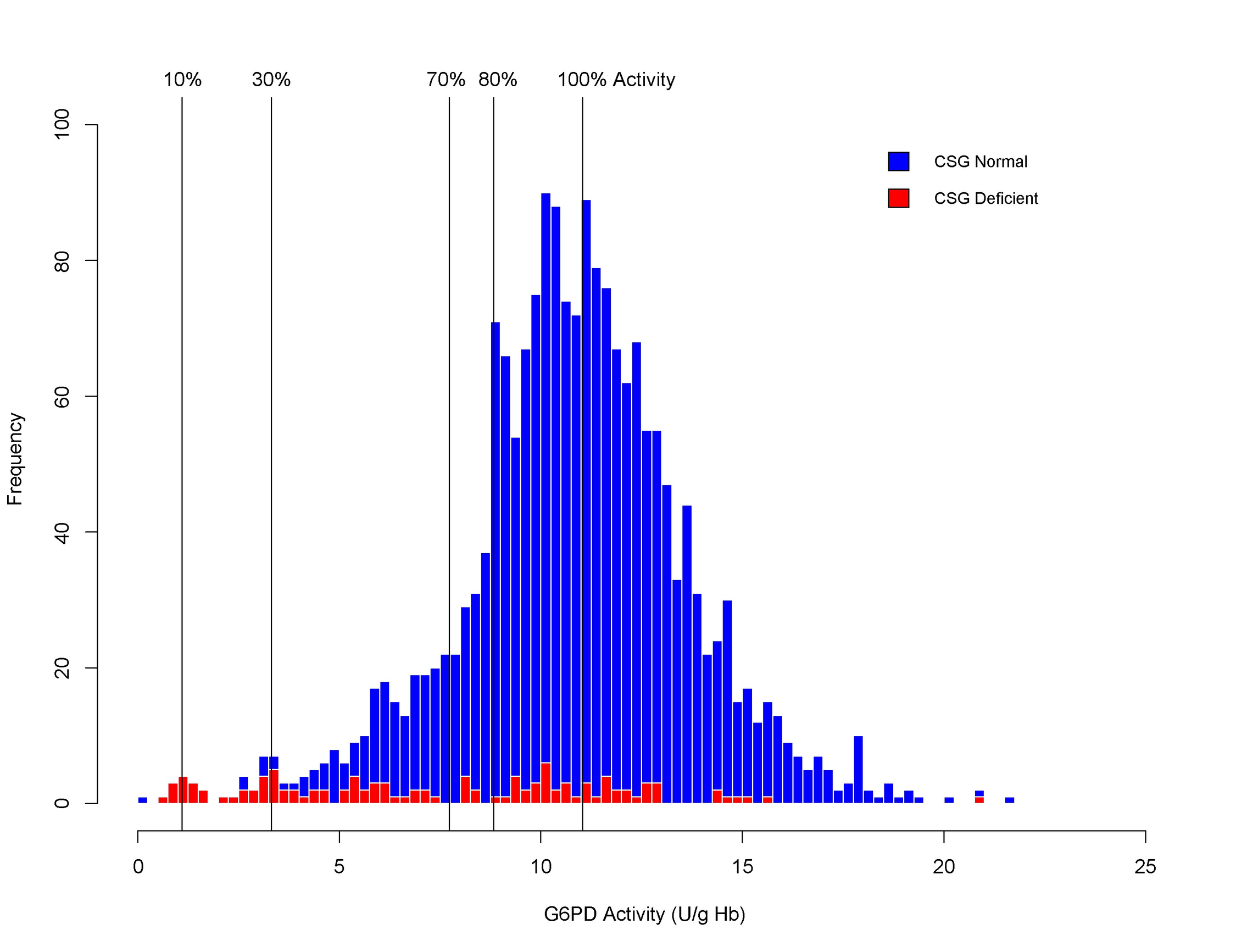

Supplement: S1 Fig — (TIF) [file pntd.0009610.s002.tif]
