# Supplementary material for: Genotypes and phenotypes of G6PD deficiency among Indonesian females across diagnostic thresholds of G6PD activity guiding safe primaquine therapy of latent malaria
Source: PLoS Negl Trop Dis. 2021 Jul 16;15(7):e0009610. doi: 10.1371/journal.pntd.0009610 (PMC8318249; doi:10.1371/journal.pntd.0009610)
Supplement: Attachment — Submitted filename: Rebuttal letter.docx [file pntd.0009610.s003.docx]

Jakarta, 17 May 2021

Dear Editors:

Dr Wuelton Marcelo Monteiro and Dr Mary Lopez-Perez

Thank you very much for your very constructive editorial comments and inputs for our manuscript. The reviewers have clearly given the manuscript a careful read and generous feedback. We appreciate their efforts very much and their help has allowed us to substantially improve the manuscript. Below are our point-by-point responses to the comments and concerns raised by the reviewers.

**Reviewer 1:**

- “It is difficult to understand from the title what the paper is about. Perhaps the title could be revised so as to be more informative?”
  - Thank you for the suggestion. We have revised the title to “**Genotypes and Phenotypes of G6PD Deficiency Among Indonesian Females Across Diagnostic Thresholds of G6PD Activity Guiding Safe Primaquine Therapy of Latent Malaria”**
- “Abstract: The conclusions point to the deficits of qualitative tests. Perhaps the abstract could end of a more positive note highlighting the ongoing roll-out of quantitative tests?”
  - We have added “…which may be remedied by point-of-care quantitative tests.” (See page 2 line 48)
- Intro: Please consider rephrasing the following sentence?  “This

113 evidence directly informs the distribution of risk with G6PD screening in the context of primaquine

114 therapy against latent vivax malaria, knowledge enabling rational strategies for maximizing essential

115 safe access to treatment against a highly prevalent and dangerous infection.”

- We have rephased the sentence to “This evidence directly informs the distribution of risk with G6PD screening in the context of primaquine therapy against latent malaria, and may serve to mitigate risk the G6PD heterozygotes.” (see page 5, lines 123-125).
- “Table 2: Please explain what you mean by double heterozygous? Please explain what is CSG?”
  - What we meant was compound heterozygous, there are 2 mutations in the G6PD gene of one female and we have changed it accordingly (see page 12-13 Table 2).
  - CSG stands for CareStart G6PD deficiency rapid test. We avoided “RDT” as that acronym is easily confused with those of malaria diagnosis.
- “Discussion: “The administration of tafenoquine is prohibited in 289 patients having <70% of normal G6PD activity;” “prohibited” may not be the most appropriate term? Better say “not recommended”.”
  - We have changed as suggested (see page 14, lines 337-337).

**Reviewer 2:**

- “It is unclear how the study population was selected? Kindly add details on the cross-sectional survey and how eligible females were enrolled. The authors state that only “healthy” females were enrolled. How was this determined? Was body temperature and history of fever considered?”
  - The study population was selected based on the high prevalence of G6PD deficiency in the area as well as a relatively high rate of malaria based on annual parasite rate reported by the health authorities near the time of the study. We have revised the text to clarify “healthy” (p. 6, lines 135-136).
  - Is the sample size sufficient to ensure adequate power to address the hypothesis being tested? The authors did not include a power calculation – kindly add.
    - Added. (See p. 6, lines 137-146)
  - The description of statistical methods is insufficient:
    - The definition of 100% G6PD activity is not explained in the methods, but only in the results, kindly add.
      - We agree and have substantially revised to include the rationale for 100% and the key diagnostic thresholds based on that value. (See p. 9, lines 203-208)
    - The authors have defined 100% activity as the mean activity of all females with activities above 5U/g Hb – kindly justify this method. From figure 2 I would have thought 100% activity is a bit lower, accordingly 30%, 70% and 80% need to be lower as well, the definition of 100% activity has obvious consequences for the conclusion.
      - We used those with activities ≥ 5U/g Hb for the simple and rational reason that this is the same threshold used in clinical laboratories to declare a person G6PD-normal or -deficient. The rationale for employing this cut off was based on the evidence-based belief that relatively highly prevalent G6PD heterozygosity would bias those diagnostic thresholds in a manner that diminished safety. In any event, we have checked on the reviewers concerns regarding an overly cautious definition of G6PD normal. If the cut off for normal G6PD activity is set at 10% exclusion, we calculated 100% normal at 10.81 U/g Hb and without exclusion at all that value is 10.78 U/g Hb compared to our applied (100%) at 11.04 U/g Hb. These differences do not significantly impact our analysis or conclusions. For example, the number of subjects having activities <70% are 232, 216 and 214 for >5 U/g Hb, >10% activity exclusion and no exclusion at all, respectively.
    - Rather than stating numbers of erroneously categorized individuals by Carestart RDT, kindly present sensitivity, specificity, NPV and PPV => discuss these findings in the discussion.
      - We have added those values, see p. 12, lines 286-288 and p. 14, lines 321-322.
    - There was a mean / median / max. (?) delay between sample collection and spectrophotometry and this delay will have impacted measured G6PD activity. Can the authors analyse the impact the delay has had (regression analysis) and if needed correct their findings accordingly? This may alter the discussion and conclusion.
      - There was not a significant delay in G6PD measurement. Samples were invariably kept cold (4^0^C) and analyzed less than one week after venipuncture. G6PD activity of intact red blood cells thus treated is known to be stable for two weeks or more.
- Are there concerns about ethical or regulatory requirements being met?

The authors state that “willing” females were enrolled. Can you kindly clarify how willingness to participate was documented?

- We stringently followed a protocol approved by an accredited local ethical review committee for research on human subjects. We have revised to express that informed consent was obtained. See p. 6, lines 149-150 and p. 7, lines 152-153.
- The authors state a malaria prevalence of 7%, kindly add absolute numbers, overall as well as for each species. Since participant identification is not explained it is unclear how reflective this prevalence is of the overall population.
  - We do not understand “participant identification” in this context.
  - We had put the data in absolute numbers in Table 1 (129/1928) and clarified this in the body of result (p. 9, line 2223). Also, we have put additional note under Table 1.
- The sentence in line 194 – 195 “A total of 1928 females…” needs to go before the sentence “The point of care G6PD rapid…” (line 192 – 193). Otherwise the reader is surprised that not everybody enrolled was tested
  - We have modified this accordingly (see p.9-10, lines 225-226).
- The authors state that 1814 females were G6PD normal and 113 females deficient (total = 1927), however also state that 1928 females were tested, there seems to be one result missing?
  - Yes, one sample did not have the Carestart RDT result (we have put ** notification under Table 1) (see p.10, line 232)
- If possible, provide a table with some baseline data on the study population
  - We do not understand what further baseline data is meant. What, specifically, does the reviewer believe is missing and relevant?
- Table 1 contains the row: “Age (median)” and next to it the result: “22.5 (95%CI)”. Please state median and interquartile range (IQR)
  - Accordingly revised. The median was 22.5 and IQR 24 (see Table 1).
- Table 1: Please state that all other entries refer to total number of persons
  - Table revised for greater clarity.
- The authors state that 103 females (approx. 5% of the study population) were G6PD normal by genotype but had activities of less than 80% - this could suggest that the definition of 100% is not correct (too high)? Can the authors discuss this?
  - The value of 100% in neither too high nor incorrect, as explained above.
- Are the figures (Tables, Images) of sufficient quality for clarity?

No, see comments on table 1 above, replace contents of table 2 with standard performance indicators (and 95%CI) and prevalence at the respective cut offs.

- Please see Supplementary Data 1.
- Are the conclusions supported by the data presented?

No, the authors base their conclusions on a questionable definition of 100% G6PD activity:

- The doubts raised by the reviewer regarding our quantitative G6PD measurements should be based on cited or expressed evidence. According to widely available guides for clinical laboratories, any G6PD activity between 5.5 and 20.5U/g Hb should be considered normal. A quick search of similar surveys of females reveals mean G6PD activities ranging between 7 and 12. We argue our definition of 100% normal G6PD activity is well within expected values and is by no means questionable. It is unclear if the reviewer bases the poor assessment of the experimental core of our study on hunch and instinct, or definitive experience and evidence. We could perhaps benefit from understanding the factual basis of this assessment.
- There was a delay of up to 3 hours before samples were introduced to a 4^o^C environment, G6PD activity deteriorates at room temperature (specifically in the tropics) rapidly and this delay will have introduced a significant bias, specifically if the delay varied. Delay to testing was up to 13 days and this will have further affected measuredG6PD activity
  - We are unaware of the evidence the reviewer seems to have regarding the rapidity of G6PD activity deterioration at room temperature. We would benefit by access to that evidence.
  - We have nonetheless clarified this in the text (see p.7, lines 157-159) that the blood in EDTA tube was immediately put on ice and that the 3 hour interval involved transport of the samples to field lab to be stored at 4^0^C in a refrigerator. Within 3 days, the samples were transported to Jakarta and analysed on the same day. G6PD activity when stored at 4^0^C is stable up to 7 days (p. 7, lines 158-160 and lines 170-171).
- The authors conclude that point of care testing for heterozygous females within the setting is challenging if not impossible, but do not mention novel G6PD biosensors that could address this diagnostic gap. Can you kindly reflect on this?
  - G6PD biosensors have not been validated to address the gap. We rationally doubt these kits will prove practical in remote rural settings in routine practice. Further we find the cost of these tests prohibitively expensive for practice in rural Indonesia. We are cautious not to put forth unproven solutions to this very difficult and dangerous problem. Nonetheless, we have added a balanced mention of these kits (see p.16, lines 368-373).
- Are the limitations of analysis clearly described?

A limitations section is missing

- We’ve added a study limitation section (see p.17, lines 390-393).
- Introduction and discussion are quite long
  - It is a complex and nuanced topic requiring proper context and technical details.

**Reviewer 3**:

- Line 57: replace “chromosome” by “gene”
  - Done.
- Line 79: replace “case” by “species” or “cause”
  - We have rectified “case” to “cause”
- Line 96: “about 8%”: this is a global average, but very variable between populations
  - Yes, this is more or less global average in malaria endemic areas.
- Line 102: could also cite <https://journals.plos.org/plosmedicine/article?id=10.1371/journal.pmed.1002992>.
  - We have added the suggested article to our reference list.
- Line 115: “highly prevalent”: citation needed
  - We have deleted the entire sentence as suggested by Reviewer 2.
- Line 124: “annual parasite incidence..” specify whether all species
  - We have added P. falciparum and P. vivax in the sentence (see p. 6, line 135).
- Line 125: suggest “at least 6 yrs” to avoid confusion with Table 1.
  - We have changed it to “…older or equal to 6 years old..”
- Line 133: if possible, state by which authority/system microscopists were certified?
  - Our microscopist was certified using guidance for such provided by the WHO.
- Line 136: “within a few hours”: please be more specific

Line 137: “within 3 days”: specify that this is the transport (not the analyses) eg.. “then cold-transported to Jakarta within 3 days for …”

- We have modified our response to “Blood in sealed EDTA tubes was put on ice immediately after venipuncture and stored at 4^0^C within a few hours. Within 3 days these samples were transported on cold packs by air to Jakarta for the laboratory analyses detailed here.” (see p.7, lines157-159)
- Line 139: any details available on test version number? Product ID?
  - Unfortunately, no.
- Line 141: how many minutes?
  - No more than 2 minutes, the blood after mixing with EDTA, was stored on ice.
- Line 143: details on training, previous experience? Were RDTs read by single technician, or double read?
  - Our technicians had previous experience with using Carestart RDT. Refresher training was done a couple of days before the team left for field study. The RDT was read by a single technician and only read by each other in the event of difficult readings where the other technician and the principal investigator would also interpret the result.
- Line 145: provide citation/justification of sample integrity after 10 days for conducting enzyme activity studies
  - This is not necessary since we did G6PD quantitative spectrophotometry test within 3 days of venipuncture. Publications have shown sample integrity up to 7 days when stored at 4^0^C.
- Line 155: was spectrophotometry performed in duplicate/replicate?
  - The spectrophotometry was done in single reading.
- Line 180: suggest double or compound heterozygotes: suggest keeping consistent
  - We used compound heterozygotes. Thank you for catching this one for us.
- Line 193: specify G6PD rapid test (not previously referred to RDT)
  - We used Carestart G6PD RDT.
- Table 1: include units; specify that pos/neg for malaria is by microscopy
  - All the numbers are in persons except for age which was in years. We also have put in note that malaria diagnostics were done by microscopy only.
- Line 207: did authors test this? Visual assessment suggests may have been more Pv among those with >100% activity
  - Yes, Pv infects more females with G6PD activity >100% however, when compared with activity < 100%, the difference is not significant.
- Line 209: confirm figures, Fig 1 suggests n=374?
  - Yes n=374.
- Line 210: “vanua lava and viangchan variants co-dominated”: possible to quantify?
  - Yes. There are 143 Vanua Lava and 98 Viangchan.
- Line 214: “lower end of G6PD activity”: perhaps could say <3 Ug/Hb?
  - It is actually less than 2 U/g Hb.
- Line 229: could provide more detail on the outliers in the Methods
  - We have used scattered boxplot to show the distribution of G6PD activity across genotypes (p. 9, lines 215-217 and p. 11, lines 262-264 - Figure 3 Legend).
- Line 235: Fig 4 shows up to 80% (not 70%)
  - Yes, this was done on purpose because we wanted to show that the trend for heterozygosity is decreasing after 70% though there were still few heterozygous females in this range. This is to further prove that 70% as cut off is adequate.
- Line 233: could specify “among the 251 heterozygotes” with <80% normal activity.
  - We have changed this as suggested except for the number of subjects who fell into this cut off were 367 females not 251.
- Line 250: interesting result that could be mentioned is the cases with <30% activity not identified by the CSG. Could consider including another histogram like fig 4 to plot out the CSG results against activity levels.
  - We have prepared the histogram as suggested. There is one sample that is supposedly very deficient by reference test but normal by RDT as seen in the histogram. Please note that RDT normal is blue and RDT deficient is red (see Supplementary Data 2).

- Table 2: suggest replacing “diagnostic performance” with “results”. Performance might be expected to include estimates of specificity, sensitivity etc. Could also include the >80% category in this table?
  - Yes, we have changed performance to diagnostic results. We could not include >80% since we did not genotype the samples beyond 80%. However, we have also prepared sensitivity, specificity etc in Supplementary Data 1.
- Paragraph from Line 274: could refer to the portable quantitative biosensor which has shown promising performance
  - Thank you for the suggestion and we have revised accordingly (see p.16, lines 370-373).
- Line 280: “increasingly insensitive”: the RDT is developed with an analytical threshold around 30%; individuals >30% activity are not intended to be identified by this assay
  - Noted. We just wanted to show that CSG (RDT) is not appropriate to screen females with activities between 30-80%.
- Discussion: occasional mention of the variant “class”: not sure what value specifying these classes adds, but may cause confusion/require some introduction for unfamiliar readers. Aren’t referred to elsewhere in the paper.
  - We have added 1 sentence to clarify this in the Introduction (p. 5, lines 106-108).
- Figure 1: “quantitative” – check spelling in 3rd box down; suggest extending the flowchart to present the genotyping and subsequent seq steps to illustrate the ND category
  - We have rectified the spelling mistake and we have also extended the flowchart beyond ND category (Figure 1).
- Figure 3: really interesting figure! Suggest including the 80% cut-off line to emphasise that the plot represents only results below this levels. Could also add N values to the x-axis labels
  - We have modified Figure 3 as suggested.
- Fig 4: Another great figure. Also suggest adding 80% activity line. Could consider including a dotted/hash overlay to the homo/compound variants so that they are more clearly differentiated from the hetero samples?
  - We have modified as suggested (see Figure 3)
- Line 400 (ref 18): “eeficiency”
  - We have rectified this. Thank you for spotting this mistake.
- Variable spectrophotometry across laboratories has meant that relative activities (as percentages) rather than absolute G6PD levels have been used to define thresholds for normal vs. deficient activity, e.g. with eligibility for tafenoquine set to 80% of normal levels. A widely adopted convention for defining 100% activity is the “Adjusted Male Median”, which is based on male activity levels. Readers who do not review the Methods section of this paper will assume that this is what was followed here too. Given that this study screened only females, the 100% level was defined as the mean activity among those with >= 5U/gHb. Given the influence that this 100% value has on the study’s analysis and conclusions (e.g. advantages of 70% vs 80%), the authors should provide more rationale and justification (How was 5U/gHb selected? How does the 100% absolute value compare to previously defined AMMs from this lab/population?) Authors should discuss the implications of this definition, and whether their conclusions regarding the 70 vs 80% cut-off would apply more broadly. The limitations of this system of defining study-specific relative thresholds could be discussed. In the Discussion, consider specifying “threshold of 70% of normal, as defined in this study, …” (line 262).
  - As the reviewer pointed out, the conventional means of arriving at what is 100% of normal employs only male populations. We could not do that. We employed the 5U/g Hb cut-off for the simple reason this is the value clinical laboratories are instructed to identify G6PD-deficiency phenotype in patients (as discussed above). We are confident of this pragmatic and reasoned approach. We have revised quite a bit in order to make this point more clearly (see p. 9, lines 207-209, p. 13-14, lines 301-312).
  - We also checked for the mean for G6PD activity at 10% exclusion (mimicked AMM) is 10.81 U/g Hb and without 10% exclusion at all is 10.78 U/g Hb compared to our current mean (100%) at 11.04 U/g Hb. Thus, the number of subjects having activities <70% are 232, 216 and 214 for >5 U/g Hb, >10% activity exclusion and no exclusion at all respectively are not significantly different.
- Discussion of the Carestart RDT performance should be in the context of the test’s analytical threshold being around the 30% level. It is designed to not distinguish 30-80% from >80% individuals. (e.g. lines 106-107, 280).
  - We have added this view in our discussion, though using the perhaps more proper “not designed to distinguish”.
- Introduction should discuss the clinical risks of radical cure to heterozygotes with 30-80% activity levels.
  - In the few studies published, some showed a drop of 30% in hematocrit in the first 5 days of treatment with PQ (Chu et al, 2017) and some heterozygous females also experienced hemolytic crises as severe as homozygous females during dapsone treatment (Pamba et al, 2012). (see p. 5, lines 117-120).
- Methods: how were study participants recruited? Were genetically related individuals excluded? (e.g. sisters or mother/daughters?)
  - The participants were socialized of the study by local health officers from nearest health centers and by village elders during church, and those who came to our study could be genetically related since there are a lot of intermarriages in the area. Some who came are mother/daughters as well.
- Results: I don’t think the authors report on whether any other variants beyond those in the SNP panel were identified by the gene sequencing. E.g. could just specify if these results will be published elsewhere.
  - We did not see other variants other than the ones identified in this manuscript, though we had hoped to discover new variants in the region.

We hope we have addressed all the comments, suggestions and revisions recommended by the reviewers. Thank you once again for these comments and hopefully it is satisfactory.

Sincerely,

Dr. sc. hum. Ari Satyagraha
